# Donor lymphocyte infusion combined with azacitidine after allogeneic HSCT in pediatric AML: a single-center retrospective analysis

**DOI:** 10.3389/fphar.2025.1727492

**Published:** 2026-01-08

**Authors:** Ana Maria Bica, Andra Daniela Marcu, Cristina Georgiana Jercan, Iuliana Iordan, Andreea Nicoleta Serbanica, Irina Avramescu, Matei Colita, Delia Codruta Popa, Ileana Constantinescu, Alexandra Mihaela Ichim, Andrei Colita, Anca Colita

**Affiliations:** 1 Faculty of Medicine, University of Medicine and Pharmacy Carol Davila, Bucharest, Romania; 2 Pediatric Bone Marrow Transplantation Unit, Fundeni Clinical Institute, Bucharest, Romania; 3 Fundeni Clinical Institute, Bucharest, Romania; 4 Department of Hematology, Coltea Hospital, Bucharest, Romania

**Keywords:** donor lymphocyte infusion, azacitidine, pediatric acute myeloid leukemia, hematopoietic stem cell transplantation, graft-versus-leukemia, graft-versus-host disease

## Abstract

**Introduction:**

Donor lymphocyte infusion (DLI) can enhance graft-versus-leukemia (GvL) effects following allogeneic hematopoietic stem cell transplantation (HSCT) in pediatric acute myeloid leukemia (AML). However, the optimal integration of azacitidine (Aza) with DLI in children remains uncertain.

**Methods:**

We retrospectively analyzed 16 pediatric AML patients (≤18 years) treated at Fundeni Clinical Institute between 2016 and 2024 who received DLI in combination with azacitidine (75 mg/m^2^/day for 7 days every 4 weeks) after HSCT. DLI was administered prophylactically or preemptively based on mixed donor chimerism (MDC), measurable residual disease (MRD) positivity, or high-risk cytogenetics, or therapeutically for post-transplant relapse, with or without chemotherapy. Outcomes assessed included overall survival (OS), donor chimerism, relapse rate, and graft-versus-host disease (GVHD).

**Results:**

After a median follow-up of 46.5 months, five patients received prophylactic/preemptive DLI and eleven received therapeutic DLI (seven with chemotherapy, four without). All patients in the prophylactic/preemptive group achieved full donor chimerism and MRD negativity, with an OS of 80% at 2.7 years. In the therapeutic group, median OS was 23.8 months with chemotherapy and 13.8 months without. OS differences between groups were not statistically significant (p = 0.384). Acute GVHD occurred in two patients (12.5%) in the therapeutic + chemotherapy subgroup; no chronic GVHD or non-relapse mortality was observed.

**Conclusion:**

Azacitidine combined with DLI is feasible and safe in pediatric AML after HSCT, particularly when applied prophylactically or preemptively to restore donor chimerism or eradicate MRD. Therapeutic use in overt relapse remains challenging and provides limited benefit. Prospective multicenter studies are needed to define optimal timing, dosing, and combination strategies for integrating azacitidine with DLI in this high-risk pediatric population.

## 1 Introduction

Acute myeloid leukemia (AML) in children is a rare but aggressive hematologic malignancy, representing approximately 15%–20% of childhood leukemias. Despite advances in chemotherapy and supportive care, long-term survival remains suboptimal for high-risk or relapsed disease (Egan and Tasian, 2023; Dholaria and Savani, 2020; Chorão et al., 2023). Allogeneic hematopoietic stem cell transplant (HSCT) offers the only curative strategy for many patients, providing both a source of healthy hematopoiesis and the critical graft-versus-leukemia (GVL) effect mediated by donor immune cells (Koreth et al., 2009; Bejanyan et al., 2015; Accorsi Buttini et al., 2024).

However, relapse remains the leading cause of treatment failure after HSCT in pediatric AML, with reported rates ranging from 20% to 40% (Bader et al., 2024). Relapse post-HSCT carries a dismal prognosis, emphasizing the need for effective strategies to prevent and treat recurrence (Zuanelli Brambilla et al., 2020; Castagna et al., 2016; Cluzeau et al., 2023).

Donor lymphocyte infusion (DLI) is well characterized in adult AML, but pediatric-specific data remain sparse, particularly regarding its integration with hypomethylating agents such as azacitidine. In adults, DLI is used preemptively for mixed donor chimerism (MDC) or measurable residual disease (MRD) positivity, prophylactically in high-risk patients, or therapeutically for relapse (Kolb et al., 1990; van Rhee et al., 1997; Rettinger et al., 2011; Pulsipher et al., 2015). Azacitidine has immunomodulatory properties, promoting the expansion and functional restoration of regulatory T cells (Tregs), thereby enhancing the GVL effect while mitigating graft-versus-host-disease (GVHD) risk (Sockel et al., 2011; ClinicalTrials.gov, 2025; Loke et al., 2021). Recent pediatric reports suggest feasibility, but optimal timing, dosing, and integration strategies remain undefined (Peters et al., 2024; Craddock et al., 2016; Admiraal et al., 2017; Schroeder et al., 2023; Tan et al., 2024; Hussain et al., 2021; Bejanyan et al., 2015).

Against this backdrop, we conducted a retrospective analysis to evaluate the outcomes of pediatric patients with AML undergoing DLI combined with azacitidine after HSCT at our center.

## 2 Materials and methods

### 2.1 Study design and patients

Single-center retrospective study at Fundeni Clinical Institute, including pediatric patients (≤18 years) with AML undergoing allo-HSCT followed by DLI and Aza (2016–2024). MDC was defined as 5%–95% donor cells by short tandem repeats (STR) analysis, per EBMT guidelines (Bader et al., 2024), while measurable residual disease (MRD) was considered positive at levels above 0.01% in bone marrow, by flow cytometry. All patients were GVHD-free and off immunosuppression at DLI.

### 2.2 Transplant procedures

Donor types included matched sibling donors (MSD), matched unrelated donors (MUD), and haploidentical. Conditioning regimens were myeloablative conditioning (MAC) or reduce intensity conditioning (RIC). Grafts were predominantly peripheral blood stem cells (PBSC).

### 2.3 DLI procedures

Indications for DLI included prophylactic or preemptive use—such as MRD positivity, MDC, or high-risk cytogenetics—and therapeutic use for disease relapse. All patients received azacitidine at a dose of 75 mg/m^2^/day for 7 consecutive days every 4 weeks prior to DLI. The administered DLI doses were 1 × 10^5^/kg, 1 × 10^5^/kg, 5 × 10^5^/kg, 1 × 10^6^/kg, and 1 × 10^7^/kg CD3^+^ cells. MRD and chimerism monitoring was performed at 1 month and subsequently at 3, 6, 9, and 12 months after transplantation, as well as whenever clinically indicated. MRD and chimerism were assessed before the first DLI dose; thereafter, chimerism was evaluated prior to each DLI, while MRD was monitored every 3 months. Relapsed patients who received therapeutic DLI in combination with chemotherapy were treated using either the FLAG, GLAG-M or DCAG protocols, along with intrathecal chemotherapy and, in some cases, radiotherapy (e.g., following orchiectomy). Patients who received therapeutic DLI without preceding chemotherapy were those experiencing either very early relapse or such extensive relapse after HSCT that administering chemotherapy would have posed a life-threatening risk.

### 2.4 Outcome and follow-up

Primary outcomes were overall survival (OS) and complete donor chimerism. Secondary outcomes: GVHD and relapse. Follow-up was measured from HSCT.

### 2.5 Statistical analysis

Conducted with IBM SPSS Statistics 25. Categorical variables were compared with Chi-squared or Fisher’s exact tests. Continuous variables were assessed for normality (Shapiro–Wilk). Normally distributed data were analyzed with ANOVA; non-normally distributed data with Kruskal–Wallis. Survival analysis used Kaplan–Meier/log-rank tests. Hazard ratios were estimated by Cox regression. Correlations were assessed by Spearman rank. Statistical significance was defined as p < 0.05.

### 2.6 Results ethics

The study was conducted in accordance with the Declaration of Helsinki and approved by the Ethics Committee of Fundeni Clinical Institute (protocol code 36105, 29.08.2025). Written informed consent was obtained from legal guardians for participation and publication of de-identified data.

## 3 Results

### 3.1 Patients’ characteristics

Sixteen patients (11M:5F), median age 10 years. Donor types: 10 MUD, 3 MSD, 3 haploidentical. Grafts: 15 PBSC, 1 BM. Twelve patients received MAC, 4 RIC for haploidentical HSCT. Median follow-up: 46.5 months (range 5–84) (Table 1).

**TABLE 1:** Patients’ characteristics.

Parameter	All patients n = 16	Prophylactic/preemptive DLI n = 5	Therapeutic DLI with chemotherapy n = 7	Therapeutic DLI without chemotherapy n = 4	p value
Age – mean ± SD, years	9.19 ± 5.47	7.00 ± 6.33	8.57 ± 5.86	11.25 ± 4.50	0.713
Male sex, n (%)	11	3 (27.3%)	4 (36.4%)	4 (36.4%)	0.296
FAB classification					
M0-2	7	2 (28.6%)	3 (42.9%)	2 (28.6%)	0.954
M4-5	9	3 (33.3%)	4 (44.4%)	2 (22.2%)	
MLL status					
Positive	4	3 (75.0%)	1 (25.0%)	0 (0%)	0.081
Negative	12	2 (16.7%)	6 (50.0%)	4 (33.3%)	
Donor type					0.201
MSD	3	2 (66.7%)	0 (0%)	1 (33.3%)	
MUD	7	3 (42.9%)	2 (28.6%)	2 (28.6%)	
MMUD	3	0 (0%)	3 (100%)	0 (0%)	
HAPLO	3	0 (0%)	2 (66.7%)	1 (33.3%)	
Conditioning regimen					0.218
MAC	12	5 (41.7%)	5 (41.7%)	2 (16.7%)	
RIC	4	0 (0%)	2 (50.0%)	2 (50.0%)	
Time from HSCT to DLI – median (IQR), months	7.82 (10.54)	8.28 (7.31)	7.36 (26.63)	7.66 (16.84)	0.663
No. of DLI – median (IQR)	4 (1)	5 (3)	4 (1)	4 (3)	0.356
Pre-DLI chimerism– median (IQR)	87.5% (34%)	91% (24%)	100% (27%)	61% (54.75%)	0.062
Post-DLI chimerism– median (IQR)	100% (7.75%)	100% (6%)	100% (7%)	68% (74.50%)	0.231
Post-DLI acute GVHD					
No	14	5 (37.5%)	5 (37.5%)	4 (28.6%)	0.230
Yes	2	0 (0%)	2 (100%)	0 (0%)	

Abbreviations: DLI, donor lymphocyte infusion; FAB, French-American-British; HAPLO, haploidentical donor; HSCT, hematopoietic stem cell transplantation; GVHD, graft-versus-host disease; MAC, myeloablative conditioning regimen; MMUD, mismatched unrelated donor; MSD, matched sibling donor; MUD, matched unrelated donor; RIC, reduced-intensity conditioning regimen; SD, standard-deviation.

### 3.2 Prophylactic/preemptive DLI

Five patients (3M/2F) received prophylactic/preemptive DLI. Median time to first DLI was 280 days. All achieved 100% donor chimerism and MRD negativity. OS at 2.7-years follow-up was 80%.

### 3.3 Therapeutic DLI (n = 11)

7 received chemotherapy prior to DLI, 4 did not. Median OS: 23.8 months (with chemotherapy) vs. 13.8 months (without). Two cases of acute GVHD (skin grade II, later progressing to gut/liver grade IV) occurred only in the chemotherapy group. No chronic GVHD or non-relapse mortality observed.

### 3.4 Statistical analyses

We performed both univariate and multivariate analyses to identify variables of potential statistical significance within our patient cohort.

### 3.5 Pre - and post-DLI chimerism

**FIGURE 1:**
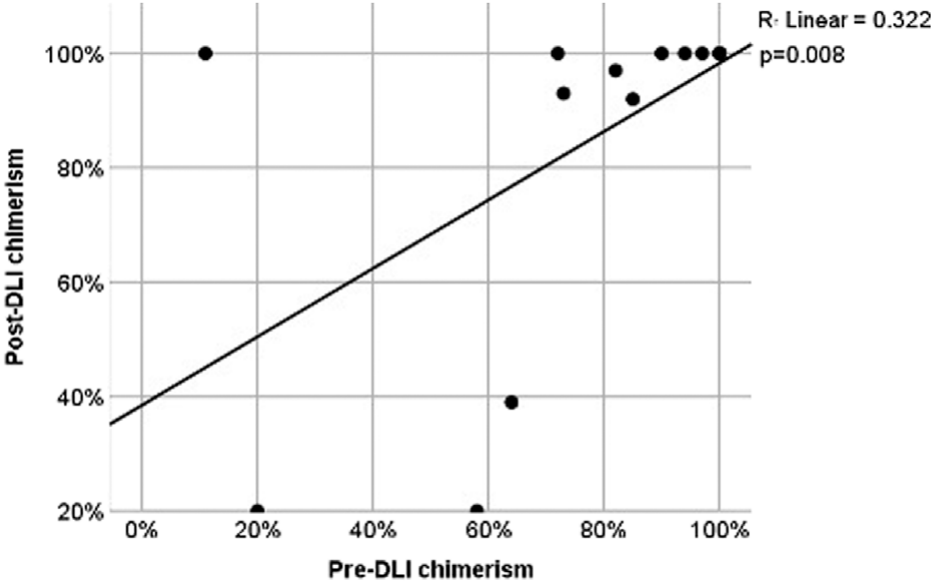
Scatter plot for pre- and post-DLI chimerism. Comparisons of OS and PFS were performed among three groups according to DLI indications: prophylactic/preemptive, therapeutic without chemotherapy, and therapeutic with chemotherapy.

The correlation between chimerism levels before and after DLI was positive and moderate-to-strong (r = 0.640). However, this correlation may reflect both baseline variability and treatment-related changes, and therefore should be interpreted with caution. This association was statistically significant (p = 0.008), indicating that higher pre-DLI chimerism values tended to be associated with higher post-DLI chimerism values (Figure 1).

**FIGURE 2:**
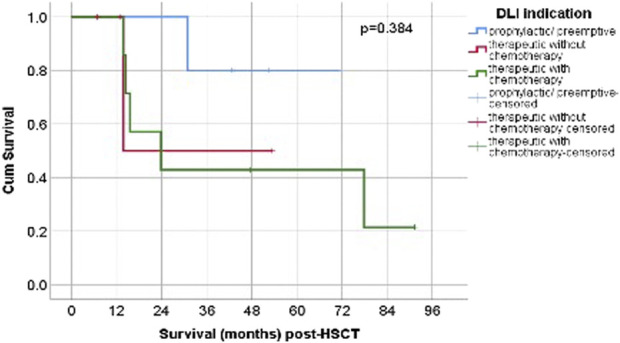
Post-HSCT OS according to DLI. Abbreviations: p/p = prophylactic/preemptive; n = number; chemo- = without chemotherapy, chemo+ = with chemotherapy.

The Kaplan–Meier curves (Figure 2) show that patients in the prophylactic/preemptive group maintained higher cumulative survival probabilities over time compared to the therapeutic groups, particularly after the first 20 months post-HCT.

The log-rank (Mantel–Cox) test revealed no statistically significant difference in survival distributions among the three groups (*p* = 0.384). While the graphical trends suggest clinical differences in long-term outcomes, these were not statistically confirmed, possibly due to limited sample size, variability in follow-up, and the proportion of censored cases.

The median survival time after DLI for the therapeutic without chemotherapy group, was 13.81 months (95% CI could not be estimated, 3/4 censored), and for the therapeutic with chemotherapy group, the median was 23.84 months (95% CI 2.49-45.18, 2/7 censored). The median OS for prophylactic/preemptive was not reached during follow-up.

**FIGURE 3:**
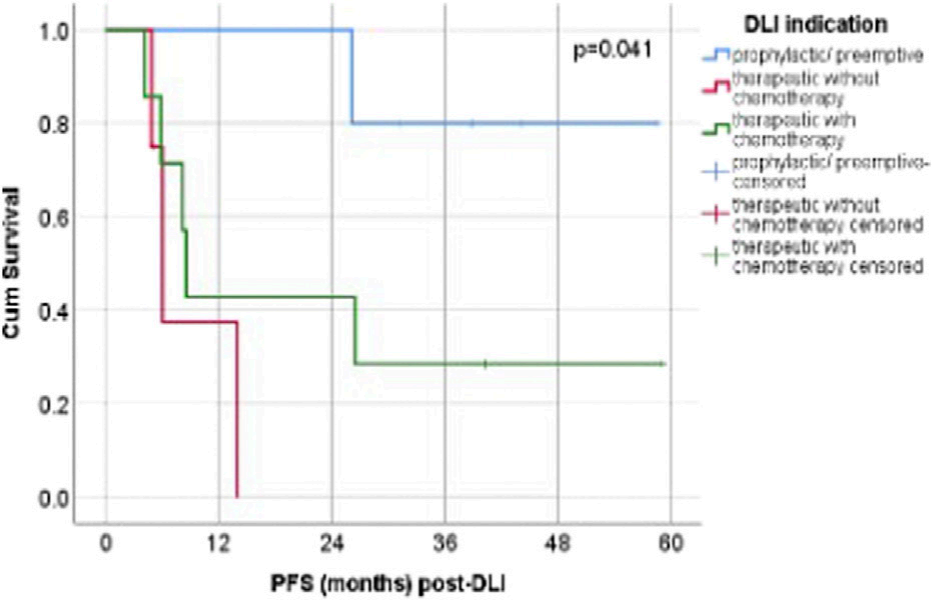
Post-DLI PFS according to DLI. Abbreviations: p/p = prophylactic/preemptive; n = number; chemo- = without chemotherapy, chemo+ = with chemotherapy.

The highest median OS was observed in the prophylactic/preemptive group and was not reached during follow-up. Among the therapeutic groups, the highest median OS was seen in patients who received chemotherapy (23.84 months, 95% CI 2.49-45.18 months), compared to without chemotherapy (13.81 months, 95% CI could not be estimated) (p = 0.384) (Figure 3). Although this difference did not reach statistical significance, patients receiving prophylactic/preemptive DLI demonstrated better survival, particularly beyond the first year post-HSCT. This lack of statistical significance may be related to the limited sample size, variability in follow-up, and the proportion of censored cases.

The Kaplan–Meier curves (Figure 3) demonstrate a visible separation between the three indication groups, with the prophylactic/preemptive group maintaining the highest probability of survival over time, followed by the therapeutic + chemotherapy group, and lastly the therapeutic without chemotherapy group. In the prophylactic/preemptive cohort, survival probability remained relatively high and stable for an extended period. The curve’s plateau indicate that a substantial proportion of patients are alive and disease-free at the last follow-up. The therapeutic without chemotherapy group displayed an earlier drop in survival probability, reflecting poorer outcomes with DLI alone. The therapeutic + chemotherapy group showed intermediate outcomes, with an initial decline in survival probability but a more gradual slope thereafter, reflecting effective disease debulking prior to DLI in some cases.

Despite these differences, the log-rank test confirmed that the observed separation was not statistically significant (*p* = 0.384). Nevertheless, the pattern supports the hypothesis that earlier prophylactic/preemptive DLI may be associated with improved survival compared with therapeutic use in overt relapse.

Regarding progression-free survival (PFS), patients who received prophylactic or preemptive DLI did not reach a median PFS (not estimable). Among patients treated therapeutically without concomitant chemotherapy, the median PFS was 6.05 months (95% CI 4.28–7.82; 1/4 censored), whereas in the group receiving therapeutic DLI combined with chemotherapy, the median PFS was 8.55 months (95% CI 7.54–9.56; 2/7 censored).

A Cox proportional hazards regression analysis was conducted to evaluate the influence of individual variables on the hazard of death or disease relapse, providing an estimate of their relative risk while accounting for time-to-event data.

In the Cox regression analysis (Table 2), the occurrence of acute graft-versus-host disease (GVHD) following donor lymphocyte infusion (DLI) did not demonstrate a statistically significant association with survival outcomes. Patients who developed acute GVHD had a hazard ratio (HR) of 1.640 (95% CI, 0.338–7.943; p = 0.539) compared with those who did not. The confidence interval was wide and crossed 1.0, reflecting substantial variability in the estimate and suggesting that any potential effect of acute GVHD on survival is uncertain. These results imply that, in this cohort, the development of acute GVHD post-DLI did not have a measurable or consistent impact on overall prognosis and should be interpreted cautiously, particularly given the likely influence of limited sample size.

**TABLE 2:** Univariate Cox Regression Analysis of Factors Affecting Outcome After DLI. Abbreviation: DLI, Donor Lymphocyte Infusion, HR, Hazard Ratio, CI, Confidence Interval, FAB, French-American-British classification, RIC, Reduced Intensity Conditioning, MAC, Myeloablative Conditioning, GVHD, Graft-versus-Host Disease.

Parameter	Univariate analysis	
	HR (95% CI)	p value
Sex		
F	Reference	
M	6.084 (0.753-49.177)	0.090
Age	1.143 (0.979-1,335)	0.091
DLI indication		
Prophylactic/preemptive	Reference	
Therapeutic without chemotherapy	14.321 (1.309-156.710)	0.029
Therapeutic with chemotherapy	5.992 (0.694-51.738)	0.104
FAB classification		
M0-2	Reference	
M4-5	0.855 (0.228-3.215)	0.817
MLL status		
Negative	Reference	
Positive	0.295 (0.036-2.385)	0.252
Conditioning regimen		
RIC	Reference	
MAC	0.211 (0.046-0.965)	0.045
Acute GVHD post-DLI		
No	Reference	
Yes	1.640 (0.338-7.943)	0.539

MLL rearrangement status was not associated with a statistically significant effect on survival. Patients with MLL-positive disease had a hazard ratio (HR) of 0.295 (95% CI, 0.036–2.385; p = 0.252) compared with MLL-negative patients. Although the point estimate suggests a possible reduction in risk, the wide confidence interval crossing 1.0 reflects considerable uncertainty, indicating that the result is not statistically reliable. These findings suggest that MLL rearrangement did not exert a consistent influence on survival and should be interpreted cautiously, particularly in light of the limited sample size.

In the Cox regression analysis, sex did not emerge as a statistically significant predictor of survival. Males had a hazard ratio (HR) of 6.084 (95% CI, 0.753–49.177) compared with females, with a p-value of 0.090. Although this indicates a possible trend toward higher mortality risk in males, the association did not reach conventional levels of statistical significance (p < 0.05). The very wide confidence interval suggests substantial variability in the estimate, most likely attributable to the limited sample size or small number of events. Accordingly, while the data may hint at a potential sex-related difference in outcome, this finding should be interpreted with caution and regarded as hypothesis-generating rather than conclusive.

In univariate analysis, the indication for donor lymphocyte infusion (DLI) was significantly associated with the studied outcome. Patients who received therapeutic DLI without prior chemotherapy had a markedly increased hazard compared to those receiving prophylactic or preemptive DLI, with a hazard ratio (HR) of 14.321 (95% confidence interval [CI], 1.309–156.710; p = 0.029), indicating a statistically significant higher risk. These results indicate that the type of DLI indication may substantially influence outcomes.

## 4 Discussion

Our retrospective analysis of pediatric AML patients undergoing DLI with azacitidine after HSCT demonstrates encouraging results, particularly in the prophylactic/preemptive setting, where we observed an overall survival (OS) of 80% and durable full donor chimerism. In the therapeutic DLI group, outcomes were less favorable, reflecting the well-documented challenges of treating overt relapse post-HSCT.

These findings are consistent with previous pediatric and adult studies that have explored the role of DLI, often in combination with hypomethylating agents, for relapse prevention or treatment after allogeneic transplantation.

For instance, Rettinger et al. (2017) showed that preemptive immunotherapy guided by chimerism could prevent relapse in pediatric AML, reporting sustained remissions in patients with early interventions. Similarly, Bellucci et al. (2002) demonstrated that prophylactic DLI could enhance immune reconstitution and sustain remission in multiple myeloma, establishing a mechanistic precedent that has since been explored in AML.

The combination of azacitidine with DLI has gained traction due to its potential to mitigate T-cell exhaustion and enhance the GVL effect without significantly increasing GVHD risk. Li et al. (2022), in a meta-analysis, concluded that this approach is effective in patients with relapsed AML and MDS after HSCT, aligning with our observation that azacitidine plus DLI is well tolerated and achieves complete donor chimerism in a substantial proportion of patients.

In the pediatric setting, Huschart et al. (2021) reported the feasibility and safety of azacitidine with prophylactic DLI in high-risk pediatric AML, similar to our cohort’s favorable outcomes in the preemptive group. More recently, Booth et al. (2023) highlighted improved survival and bone marrow T-cell repertoire diversification with azacitidine plus prophylactic DLI, underscoring the immunologic rationale behind this approach.

Our therapeutic DLI group, where patients received DLI primarily for frank relapse, had an OS of 45.4%, mirroring outcomes reported by Kharfan-Dabaja et al. (2018) who found comparable long-term survival between DLI and second HSCT in adult AML patients. However, our data emphasize the superior outcomes achieved when DLI is used preemptively rather than in overt relapse, consistent with findings from Guillaume et al. (2021) and Caldemeyer et al. (2017) who reported higher long-term survival rates in mixed-chimeric or high-risk populations treated preemptively.

GVHD remains a concern with DLI, particularly after multiple infusions or in the setting of unrelated/haploidentical donors. In our study, significant GVHD developed in only two patients (12.5%), which is comparable to the incidence rates of 10%–20% reported in large series (e.g., Suryaprakash et al., 2023; Yang et al., 2023), suggesting that careful patient selection, monitoring, and concurrent azacitidine may mitigate GVHD risk.

Finally, the emergence of FLT3 inhibitors and novel immunotherapeutics offers future avenues for combination with DLI. As noted by Najima (2023) and Kreidieh et al. (2022), integrating targeted agents may further improve outcomes, especially in FLT3-mutated cases, a subgroup also represented in our cohort.

Donor lymphocyte infusion in pediatric AML is a fairly niche area and most publications are case reports, small case series, or retrospective registry analyses, because DLI is more studied in adult AML or in pediatric acute lymphoblastic leukemia/relapse settings. In Table 3 is a list of peer-reviewed articles specifically addressing DLI in pediatric AML, including mixed chimerism or progressive mixed chimerism.

**TABLE 3:** Selected studies of DLI in pediatric AML.

*Study*	*No of patients*	*Indication of DLI*	*Survival rate*	*Notes*
Bader et al. (2004)	81	Prophylactic (CC, MC)	52% EFS at 3 years	GvHD, TRM Multicenter trial
Rettinger et al. (2011)	71	Preemptive		GvHD (75%) Multicenter trial
Gefen et al. (2013)	2	Therapeutic	0%	GvHD, AML subset
Rujkijyanont et al. (2013)	16	Prophylactic (MC)		GvHD, AML subset
Gozdzik et al. (2015)	11	Prophylactic, preemptive, therapeutic		GvHD, AML subset
Huschart et al. (2021)	11	Prophylactic	100%	GvHD (10%), mFU 17months
Booth et al. (2023)	17	Prophylactic	88.2%	GvHD

In this cohort of studies examining donor lymphocyte infusion (DLI) across various indications, both prophylactic and therapeutic approaches demonstrate heterogeneous outcomes with respect to survival and graft-versus-host disease (GvHD) incidence. Bader et al. (2004) reported a 52% event-free survival (EFS) at 3 years in a multicenter trial of 81 patients receiving prophylactic DLI and more recent analyses, such as Huschart et al. (2021) and Booth et al. (2023), demonstrated high survival rates (up to 100% and 88.2%, respectively) with prophylactic DLI, accompanied by relatively low GvHD incidence, highlighting potential improvements in patient selection, dosing strategies, and supportive care over time. Collectively, these studies illustrate that while prophylactic and preemptive DLIs can confer survival benefits, their statistical impact on GvHD incidence and overall outcomes is strongly influenced by timing, patient population, and sample size, emphasizing the need for larger, controlled studies to determine optimal DLI strategies.

This study represents one of the largest pediatric single-center series evaluating DLI combined with azacitidine post-HSCT. We observed durable remission and chimerism correction with prophylactic/preemptive DLI, while therapeutic DLI for overt relapse remained less effective.

Our findings align with prior pediatric series (Rettinger et al., 2017; Huschart et al., 2021; Booth et al., 2023), highlighting the feasibility and safety of azacitidine + DLI, particularly in the preemptive setting. Adult studies (Loke et al., 2021; Craddock et al., 2016) also support its role in relapse prevention, though outcomes in overt relapse remain modest. The low incidence of GVHD in our cohort suggests that azacitidine may help balance GVL and GVHD.

This study has several limitations. Its retrospective design carries an inherent risk of selection bias and unmeasured confounding. The small sample size and absence of a control group reduce statistical power and increase the likelihood of type II error. In addition, the limited number of events did not allow for reliable multivariable modeling. These factors should be taken into account when interpreting the results. Larger prospective multicenter studies are required to optimize timing, dosing, and integration with novel agents (e.g., FLT3 inhibitors, venetoclax).

## 5 Conclusion

In summary, our findings indicate that the combination of azacitidine and donor lymphocyte infusion is safe and feasible in pediatric AML following allogeneic HSCT, with the most favorable outcomes seen when applied prophylactically or preemptively to stabilize donor chimerism and control MRD. While the data point toward a potential clinical benefit, the study was not powered to establish definitive efficacy, and the results should be interpreted in light of the cohort’s limited size and inherent heterogeneity.

## Data Availability

The raw data supporting the conclusions of this article will be made available by the authors, without undue reservation.
